# Risk factors and outcomes of diabetic foot ulcer among diabetes mellitus patients admitted to Nekemte referral hospital, western Ethiopia: Prospective observational study

**DOI:** 10.1016/j.amsu.2020.01.005

**Published:** 2020-01-18

**Authors:** Firomsa Bekele, Legese Chelkeba, Ginenus Fekadu, Kumera Bekele

**Affiliations:** a Department of Pharmacy, College of Health Science, Mettu University, Mettu, Ethiopia; b Department of Pharmacy, Institute of Health Science, Jimma University, Jimma, Ethiopia; c Department of Pharmacy, Institute of Health Science, Wollega University, Nekemte, Ethiopia; d Department of Nursing, College of Health Science, Selale University, Fiche, Ethiopia

**Keywords:** Diabetic foot ulcer, Outcomes, Risk factors, Nekemte, Ethiopia

## Abstract

**Objectives:**

Diabetic foot ulcer is one of the complications of diabetes mellitus. The diabetic patients with foot infections especially gangrene require long-term hospitalization and carry the risk of limb amputation. Despite these challenges, there are a scarce studies done on risk factors and no finding on outcomes of diabetic foot ulcers in Ethiopia.

**Patients and methods:**

A prospective observational study was conducted among diabetes patients with diabetic foot ulcer at Nekemte referral hospital from March 15 to June 15, 2018.

**Results:**

Of the 115 diabetes foot ulcer patients admitted to Nekemte referral hospital, 64(55.65%) were males and the mean age of participants was 44.4 ± 14.7 years. Fifty-eight (50.43%) of the patients had chronic health problems and 56(48.69%) had diabetic complications. Of patients with complications, 35(30.43%) were undergone amputations. Diabetic foot ulcer grade ≥4 *(AOR* = *1.7; 95% CI: 1.604, 4.789),* inappropriate antibiotics use (*AOR* = *2.526; 95% CI: 1.767, 8.314),* overweight (*AOR* = *2.767; 95% CI: 1.827, 9.252),* obesity (AOR = 3.020; 95% CI: 2.556, 16.397), poor blood glucose control *(AOR* = *2.592; 95% CI: 1.937, 7.168)* and neuropathy *(AOR* = *1.565; 95% CI: 1.508, 4.822)* were predictors of amputation up on multivariable logistic regression analysis.

**Conclusion:**

Blood glucose level, higher body mass index, inappropriate antibiotics use, neuropathy and advanced grade of diabetic foot ulcer were independent predictors of amputation. Thus, special emphasis for patients having neuropathy and advanced grade of diabetic foot ulcer as well as weight reduction, managing hyperglycaemia, and appropriate antibiotics prescription practice would decrease untoward effects of diabetic foot ulcer.

## Highlights

• Foot problems are common in people with diabetes throughout the world, affecting up to 15% of diabetic patients.
• Diabetic foot complications is the major medical, social and economic problem for all types of diabetes.
• Advanced diabetes foot infections are the most common diabetes-related cause of hospitalization.
• Empiric antibiotic regimen is selected for diabetic foot infections on the basis of the likely etiologic agent(s).
• Peripheral arterial disease (PAD) is present in one-half of all patients with foot ulcers.

## 1 Background

Diabetes mellitus (DM) is a non-communicable disease and one of the most common chronic diseases [1]. World health organization defined DM as a metabolic disorder of multiple etiology characterized by chronic hyperglycemia with disturbances of carbohydrate, fat and protein metabolism resulting from defects in insulin secretion, insulin action, or both [2]. Complications of DM become a major public health problem in all countries [3]. It is characterized by multiple long-term complications affecting almost every system in the body and often leads to blindness, heart and blood vessel disease, stroke, kidney failure, amputations, and nerve damage [4].

Diabetic patients who present with foot ulceration are associated with many risk factors. Peripheral arterial disease (PAD) is present in approximately one-half of all patients with foot ulcers and considered an important predictor of outcome [5]. Patients in whom their foot ulcer progressed to diabetic foot infections not only suffer from prolonged hospitalization but also leads to amputations of their foot which increases the rates of mortality [6]. Along with increased morbidity, foot ulcers can lead to lifelong disability and substantially diminish the quality of life (QOL) for these patients. Specifically, patients with diabetic foot ulcer (DFU) have restrictions on mobility, poor psychosocial adjustment, and lower self-perceptions of health than patients who do not have ulcers. The survival rate of patients with diabetic foot ulcer was decreased compared to diabetes patients without foot ulcer [7,8].

DM has been established as one of the most common and important disease states associated increased risk of postoperative infections and poor outcomes after lumbar spinal surgery [9]. DM patients undergoing degenerative cervical spine surgery also have an increased risk for several preoperative complications [10]. Foot problems remain very common in people with diabetes throughout the world, affecting up to 15% of diabetic patients during their lifetime [11].

Foot complications, especially foot ulcers, constitute a major public health problem for diabetes patients in sub-Saharan Africa and are important causes of prolonged hospital admission and death in patients from this part of the continent [12]. Diabetes foot infection due to gangrene is the most common cause of prolonged hospitalization and amputation of their limbs. Besides, 28%–51% of amputated diabetics will have a second amputation of the lower limb within five years of the first amputation [13,14].

Generally, diabetic foot complications remain the major medical, social, and economic problems for all types of diabetes [11,15]. In Ethiopia, patient habits of poor foot-care practice, and the absence of good quality service of DFU may leads to foot infections which results in limb amputation [16]. Only a few pharmacists were assigned to avoid the inappropriate use of antibiotics in Nekemte referral hospital (NRH) by intervening problems at only dispensing levels. Despite these challenges, no study has been conducted on risk factor and outcomes of DFU in NRH. This study tried to identify the risk factors and outcomes of DFU patients admitted to NRH.

## 2 Patients and methods

### 2.1 Study setting, design and study period

A prospective observational study was conducted at NRH from March 15 to June 15, 2018. The hospital is located in Nekemte town, located 330 km to the west of Addis Ababa, the capital city of Ethiopia. The hospital gives health service for more than 10, 000,000 people living in western Ethiopia. This hospital serves as a referral hospital, a teaching hospital, and research center, and the hospital has one diabetic clinic. There were about 2420 diabetic patients who have been following the diabetic clinic annually. The study population and methods shares similarity with a previously published article by Bekele F et al. [17]. The work has been reported in line with the strengthening the reporting of cohort studies in surgery (STROCSS) criteria [18].

### 2.2 Study participants and eligibility criteria

Patients ≥18 years who were diagnosed as diabetes, had DFU, willing to participate in the study and had any visible foot lesions were included. Patients who had traumatic ulcers due to other than normal causes like car accident, burn and any injury due to sharpened materials and who was in acute stress were excluded.

### 2.3 Study variables and outcome endpoints

Amputation was the primary outcome and the Wagner classification of DFU was used to assess the severity of foot ulcer. Extent (size) was determined by multiplying the largest by the second largest diameter perpendicular to the first. The etiologies of diabetic foot infection were identified by gram stains. Amputation and healing status was measured using a checklist and assessed by close follow up through telephone interview of the patient/caregiver/proxy on a weekly basis.

### 2.4 Sample size and sampling technique

Single population proportion formula was used to calculate the required sample size by considering the following assumptions: n is required sample size, P is incidence of amputation which was 29%, the rate found at Muhimbili National Hospital, Tanzania [12], Z is standardized normal distribution value at the 95% CI: 1.96 and d is the margin of error of 5%.

$$\text{n}=\frac{{\left({\text{Z}}_{\frac{\alpha }{2}}\right)}^{2}\text{p}\left(1-\text{p}\right)}{{\text{d}}^{2}}$$

z = 1.96

P = 29% (0.29)

d = 0.05

$$\text{n}=\frac{{\left(1.96\right)}^{2}\;(0.29)\;(0.71)=316}{{\left(0.05\right)}^{2}}$$

The expected number of population in the study period based on the average number of patients visiting the hospital was 156. The corrected sample size, using the correction formula was 104. A 10% contingency yielded a final sample size of 115. Conveniently all patients during the study period fulfilling the eligibility criteria were included in the final analysis.

### 2.5 Data collection process and management

Data was collected using a questionnaire developed after reviewing different literatures and adopting it based on available data. One medical doctor, one nurse and one pharmacist were recruited as data collector and the data was supervised by another medical doctor. A pus swab was obtained from the ulcers before any ulcer cleaning and antibiotics given or debridement done to avoid contamination. The samples were delivered to the laboratory immediately and a thin smear was prepared on grease or oil-free slides. Appropriateness of antibiotics was identified based on standard guidelines of Infectious Diseases Society of America (IDSA) for diagnosis and treatment of diabetic foot infection [20], which is based on the most likely coverage of antibiotics for treatments of diabetic foot infection for identified gram stain and appropriateness of dosage regimens. Five percent of the sample was pre-tested to check the acceptability and consistency of data collection tool two weeks before the actual data collection. The patients were followed for consecutive three months with telephone interviews after patients were discharged from the hospital.

### 2.6 Data processing and analysis

The collected data was entered into EPI-manager 4.0.2 and analysis was done using statistical package for social sciences (SPSS) 24. Descriptive data was explained by frequency and percentage. The obtained results were explained by means and standard deviations. Logistic regression was used to analyse the variables and each variable was evaluated independently in bivariate analysis and association was determined using cross-tabulation and crudes odds ratio (COR) with 95% confidence interval (CI). All variables associated with the amputation at a probability level of less than or equal to 0.25 on the bivariate analysis were entered into a multivariate logistic regression analysis to control for confounders. The strength of association was described using adjusted odds ratio (AOR) and variables with a p-value < 0.05 had a statistically significant association with the amputation.

### 2.7 Ethics approval and consent to participate

Ethical clearance was obtained from the Institutional Review Board (IRB) of Jimma University, Institute of health with a reference number of IHRPGC/104/208. The ethics waiver was not applicable since the study was not a randomized clinical trial so that the study subjects were not divided into experimental and control groups. Permission was obtained from the medical director of the NRH to access diabetes patients and conducts the study. The benefit and risks of the study was explained and written consent was obtained from each patient involved in the study. To ensure confidentiality, name and other identifiers of patients and health care professionals were not recorded on the data collection tools. The study was registered researchregistry.com with a unique reference number of “researchregistry5237”.

### 2.8 Operational definitions

#### 2.8.1 Diabetic Foot ulcer

The foot of a diabetic patient that has the potential risk of pathologic consequences, including infection, ulceration, and/or destruction of deep tissues.

#### 2.8.2 Healing

The complete closure of the ulcer with skin intact (complete epithelialization) and without drainage or sinus formation.

#### 2.8.3 Amputation

The complete or partial removal of a limb or body appendage by surgical or traumatic means.

#### 2.8.4 Minor amputation

Amputation involving below ankle.

#### 2.8.5 Major amputation

Amputation of legs which involves above the ankle.

#### 2.8.6 Grades of diabetic's foot ulcer

For purpose of this study we used Wagner system for classification of diabetic foot ulcer which uses 6 wound grades (scored 0 to 5) to assess ulcer depth [19].

• Grade 0 diabetic foot ulcer: No ulcer, but the foot is at risk for ulceration
• Grade 1 diabetic foot ulcer: Superficial ulceration
• Grade 2 diabetic foot ulcer: Ulcer with deep infection, but without involvement of the bone
• Grade 3 diabetic foot ulcer: Ulcer with osteomyelitis.
• Grade 4 diabetic foot ulcer: Presence of localized gangrene on the foot.
• Grade 5 diabetic foot ulcer: Presence of gangrene of the whole foot.

#### 2.8.7 Neuropathy

It was diagnosed if the patient had at least one manifestation from the following list of manifestations: burning pain, vibration from the skin, gradual numbness, freezing, extremely sensitive to touch, muscle weakness, and lack of coordination.

#### 2.8.8 Peripheral vascular disease

It is an arterial and vein disease at the peripheral region, which often occurs in diabetic patients.

#### 2.8.9 Glycemic control

Categorized based on American Diabetic Association (ADA) recommendation in to two groups:

• **Good glycemic control:** Fasting blood glucose of 70–130 mg/dl.
• **Poor glycemic control:** Fasting blood glucose of <70 mg/dl and >130 mg/dl

#### 2.8.10 Appropriate drug

Antibiotics prescribed in accordance with infectious diseases society of America (IDSA) guideline for the treatment of diabetic foot infection recommendation based on gram stains and dosage regimens.

#### 2.8.11 Inappropriate drug

Antibiotics prescribed inconsistent with infectious diseases society of America (IDSA) guideline for the treatment of diabetic foot infection recommendation based on gram stains and dosage regimens.

## 3 Results

### 3.1 Socio-demographic characteristics

Over the study period, 115 DFU patients were admitted to the NRH and 64(55.65%) were males. About 26(22.61%) of them were in the age range of 58–67 and the mean age of participants was 44.4 ± 14.7 years. About 34(29.57%) of the DFU patients were overweight and 16(13.91%) were obese while the mean body mass index (BMI) was 24.94 ± 3.69 kg/m^2^(Table 1).

**Table 1:** Socio-demographic characteristics of respondents in Nekemte referral hospital, west Ethiopia, 2018.

Variables		Frequency (n)	Percent (%)
Sex	Male	64	55.65
	Female	51	44.35
Age (years)	18–27	16	13.91
	28–37	14	12.17
	38–47	15	13.04
	48–57	24	20.87
	58–67	26	22.61
	68–77	20	17.39
Marital Status	Married	80	69.57
	Single	21	18.26
	Window	8	6.96
	Divorced	6	5.22
Residence	Urban	58	50.43
	Rural	57	49.57
Educational level	Illiterate	24	20.87
	Primary school	29	25.22
	Secondary school	22	19.13
	Above Secondary school	40	34.78
BMI (kg/m^2^)	<24.5	65	56.52
	24.5–29.5	34	29.57
	>29.5	16	13.91

BMI: Body mass index NGO: Non-governmental organization.

### 3.2 Medical conditions and behavioural characteristics

A total of 58(50.43%) of the participants had foot ulcer and chronic health problems or co-morbidity with other diseases. Among these, 56(48.69%) participants had hypertension as comorbidity. Thirty (26.09%) of the study participants were current smokers and 38 (33.04%) were current alcohol drinkers (Table 2).

**Table 2:** Co-morbidities, complications and behavioural characteristics among diabetic foot ulcer patients attending the Nekemte referral hospital, west Ethiopia, 2018.

	Variables	Frequency (n)	Percent (%)
Co-morbidities and complications	Retinopathy	55	47.83
	Neuropathy	52	45.22
	Nephropathy	46	40.00
	Hypertension	56	48.69
	Peripheral vascular disease	42	36.52
	Coronary heart disease/ischemic heart disease	41	35.65
	Dyslipidaemia	40	34.78
Behavioural characteristics	Previous alcohol drinker	39	33.91
	Current alcohol drinker	38	33.04
	Previous smoker	30	26.09
	Current smoker	30	26.09

Among 115 study participants, 61 (53.04%) of them had type 2 diabetes mellitus. The mean fasting blood glucose level among diabetic patients with foot ulcer was 147.93 ± 45.03 mg/dl. Twenty-six participants (22.61%) were diabetic for more than 10 years and 53(46.09%) participants had poorly controlled blood glucose levels. Among Forty-two (54.55%) patients the isolated micro-organism was gram-positive and ulcer size greater than 5 cm^2^ was identified among 23(20.00%) patients (Table 3).

**Table 3:** Clinical characteristics of diabetic foot ulcer patients among diabetes mellitus patients admitted to Nekemte Referral Hospital, west Ethiopia, 2018.

Variables		Frequency (n)	Percent (%)
Types of DM	Type 2 DM	61	53.04
	Type 1 DM	54	46.96
Duration of DM	<5years	42	36.52
	5–10years	47	40.87
	>10 years	26	22.61
Glycaemic control	Good control	62	53.91
	Poor control	53	46.09
Size of Ulcer	<1 cm^2^	66	57.39
	1–5 cm^2^	26	22.61
	>5 cm^2^	23	20.00

About 9(7.83%) of the patients, dorsal/inter-digital toes were amputated and 4(3.48%) patients had amputation on heel (Table 4).

**Table 4:** The location of diabetic foot ulcer patients admitted to Nekemte referral hospital, west Ethiopia, 2018.

Location of ulcer	Amputation		
	Yes (%)	No (%)	Total (%)
Dorsal/inter digital toes	9(7.83)	22(19.13)	31(26.96)
Plantar forefoot/mid foot/Plantar hind foot	11(9.57)	26(22.60)	37(32.17)
Plantar toes	6(5.22)	24(20.87)	30(26.09)
Dorsal foot	5(4.35)	4(3.48)	9(7.83)
Heel	4(3.48)	4(3.48)	8(6.96)

### 3.3 Antibiotics prescribed to treat DFU

Empiric antibiotic regimens were prescribed for DFU patients after gram stain results were obtained and given based on the severity of the infection as well as the likely etiologic agent. Accordingly, an initial antibiotic course for a soft tissue infection of about 7 days for mild infections and 10–21 days for moderate to severe infections were given. Cloxacillin (34.15%) was the most commonly prescribed antibiotic for the treatment of DFU followed by metronidazole and ceftriaxone (Table 5). From the total patients’ given antibiotics, 38(49.35%) of them prescribed appropriately and 39(50.65%) were prescribed inappropriately.

**Table 5:** Commonly prescribed individual antibiotics for treating diabetic foot ulcer in Nekemte referral hospital, west Ethiopia, 2018.

Antibiotics	Frequency (n)	Percent (%)
Cloxacillin	56	34.15
Metronidazole	43	26.22
Ceftriaxone	33	20.12
Ampicillin	9	5.49
Chrompenicol	8	4.88
Gentamycin	5	3.05
Ceftazidime	4	2.44
Ciprofloxacillin	3	1.83
Vancomycin	2	1.22
Amoxacillin	1	0.61
Total	164	100

### 3.4 Risk factors and outcomes of diabetic foot ulcer

From the patients who developed DFU, 80(69.57%) were healed and 35(30.43%) of them were amputated. From those amputated DFU patients, 20(57.14%) and 15(42.86%) were undergone minor and major amputation, respectively. From the patients who undergone major amputation, 9(60%) of them were amputated below the knee and 6(40%) of them were amputated above the knee. Foot ulcer grade ≥4, inappropriate antibiotics use, overweight, obesity, poor blood glucose control, and neuropathy were found to be predictors of amputation up on multivariable logistic regression analysis.

Those diabetic patients who had Grade ≥4 diabetic foot ulcer were1.7 times more likely to be amputated than those Grade < 4 diabetic foot ulcer (AOR = 1.7; 95% CI: 1.604, 4.789). Patients with diabetic foot ulcer who had taken inappropriate antibiotics were 2.5 times more likely to be amputated than those who taken appropriate antibiotics (AOR = 2.526; 95% CI: 1.767, 8.314). Overweight diabetic patients were 2.8 times more likely to undergone amputations compared to diabetic patients with normal weight (AOR = 2.767; 95% CI: 1.827, 9.252) and obese diabetic patients were 3 times more likely to undergone amputation as compared to diabetic patients with normal body mass index (AOR = 3.02; 95% CI: 2.556, 16.397). Additionally, those with diabetic foot ulcer who had poor blood glucose control were 2.6 more likely to undergone amputation as compared to diabetic foot ulcer patients who had good controlled blood glucose level (AOR = 2.592; 95% CI: 1.937, 7.168). Furthermore, those DFU patients who had neuropathy were 1.6 times more likely to undergone amputation as compared to those diabetic foot ulcer patients without neuropathy (AOR = 1.565; 95% CI: 1.508, 4.822) (Table 6).

**Table 6:** Multivariate logistic regression analysis result of factors associated with amputation among diabetic foot ulcer patients admitted to NRH, west Ethiopia, 2018.

Variables		Amputation		COR(95%CI)	P value	AOR(95%CI)	P value
		Amputated n (%)	Not Amputated n (%)				
Residence	Rural	17(29.82)	40(70.18)	1.720[0.392,7.554]	0.247*	1.547[0.364,6.579]	0.416
	Urban	18(31.03)	40(68.97)	1		1	
Sex	Male	19(29.69)	45(70.31)	1.639[0.169,2.410]	0.158*	1.660[0.178,2.451]	0.221
	Female	16(31.37)	35(68.63)	1		1	
Drinking Alcohol Currently	Yes	12(31.58)	26(68.42)	1.516[0.107,2.485]	0.240*	1.594[0.128,2.761]	0.693
	No	23(29.87)	54(70.13)	1		1	
Smoking cigarette currently	Yes	12(40)	18(60)	1.426[0.362,5.622]	0.188*	1.359[0.346,5.345]	0.222
	No	23(27.06)	62(72.94)	1		1	
Previous history of Ulcer	Yes	16(32.65)	33(67.35)	1.656[0.175,2.462]	0.235*	1.674[0.182,2.499]	0.317
	No	19(28.79)	47(71.21)	1		1	
Types of DM	Type 2 DM	23(37.70)	38(62.30)	1.483[0.117,2.001]	0.074*	1.431[0.108,1.715]	0.057
	Type 1 DM	12(22.22)	42(77.78)	1		1	
Hypertension	Yes	16(28.57)	40(71.43)	2.951[0.216,4.186]	0.167*	1.109[0.267,4.604]	0.509
	No	19(32.20)	40(67.80)	1		1	
Ischemic Heart Disease	Yes	14(34.15)	27(65.85)	1.184[0.298,4.708]	0.152*	1.133[0.345,5.156]	0.361
	No	21(28.38)	53(71.62)	1		1	
Dyslipidaemia	Yes	13(32.50)	27(67.50)	2.645[0.141,2.937]	0.157*	1.766[0.185,3.170]	0.629
	No	22(29.33)	53(70.67)	1		1	
Retinopathy	Yes	17(30.90)	38(69.10)	1.345[0.092,1.386]	0.113*	1.358[0.097,1.319]	0.052
	No	18(30)	42(70)	1		1	
Neuropathy	Yes	20(38.46)	32(61.54)	2.658[1.561,12.602]	0.029*	1.565[1.508,4.822]	0.004**
	No	15(23.81)	48(76.19)	1		1	
Coronary Heart Disease	Yes	9(33.33)	18(66.67)	1.176[0.035,2.891]	0.078*	1.179[0.135,1.915]	0.179
	No	26(29.55)	62(70.45)	1		1	
Nephropathy	Yes	18(39.13)	28(60.87)	1.645[0.148,2.814]	0.100*	1.227[0.173,3.013]	0.604
	No	17(24.64)	52(75.36)	1		1	
Peripheral Vascular Disease	Yes	13(30.95)	29(69.05)	1.243[0.283,5.452]	0.177*	1.440[0.343,6.048]	0.864
	No	22(30.14)	51(69.86)	1		1	
Body mass index	<24.5	15(23.08)	50(76.92)	1	0.073*	1	0.122
	24.5–29.5	12(35.29)	22(64.71)	7.054[1.410,35.296]	0.032*	2.767[1.827,9.252]	0.021**
	>29.5	8(50)	8(50)	7.729[2.828,72.134]	0.017*	3.020[2.556,16.397]	0.019**
Glycaemic Control	Poor Control	21(39.62)	32(60.38)	2.779[1.755,10.231]	0.048*	2.592[1.937,7.168]	0.023**
	Good Control	14(22.58)	48(77.42)	1		1	
Duration of Diabetic Mellitus	<5years	14(33.33)	28(66.67)	1	0.184*	1	0.174
	5–10years	14(29.79)	33(70.21)	2.273[0.299,5.418]	0.071*	1.171[0.279,4.910]	0.061
	>10years	7(26.92)	19(73.08)	1.672[0.112,4.011]	0.157*	1.596[0.103,3.439]	0.558
Size of Ulcer	<1 cm^2^	16(24.24)	50(75.76)	1	0.215*	1	0.109
	1–5 cm^2^	11(42.31)	15(57.69)	1.515[0.109,2.431]	0.190*	1.608[0.139,2.665]	0.253
	>5 cm^2^	8(34.78)	15(65.22)	1.881[0.154,5.027]	0.132*	1.894[0.158,5.054]	0.130
Appropriateness of Antibiotics	Inappropriate	21(53.85)	18(46.15)	6.192[1.108,34.614]	0.012*	2.526[1.767,8.314]	0.017**
	Appropriate	14(36.84)	24(63.16)	1		1	
Grade of Ulcer	Grade <4	19(21.84)	68(78.16)	1	0.001*	1	0.005**
	Grade ≥4	16(57.14)	12(42.86)	3.209 [1.790,13.033]		1.70[1.604,4.789]	

Note *Shows statistically significant p-value ≤0.25 at 95% CI.

**Shows statistically significant p-value ≤0.05 at 95% CI.

## 4 Discussion

This study tried to assess the risk factors and outcomes of DFU patients admitted NRH, western Ethiopia. This study found that almost half of the patients had poor glycaemic control and revealed that poor blood glucose control patients were more likely to be amputated as compared with those who had good blood glucose control. This was consistent with the studies conducted in USA, Germany, India, and Sudan [9,13,21,22]. This indicates that the importance of glycaemic control should be implied and emphasized by these findings as a key aspect of primary intervention in DFU management and prevention of unnecessary limb wastage. Therefore, optimal control of plasma glucose will decrease the risk of amputation in diabetic foot ulcer patients.

The result of this study showed that overweight and obese DFU patients were 2.8 and 3 times more likely to undergone amputation as compared to those who had a normal BMI, respectively. This was consistent with the study conducted in Gondar [23]. But the study done in Kenya showed that BMI was not associated with diabetic foot ulcer [6]. The possible reason could be due to the decreased blood flow circulations to the lower limb as a result of fat accumulations among higher BMI patients.

Advanced Wagner stage ulcers were a significant risk factor for amputation. DFU patients who had Wagner Grade ≥4 were 1.7 times more likely to be amputated as compared to diabetic foot ulcer patients who had Wagner Grade <4. This result was consistent with the studies conducted in USA and Tanzania [12,24]. The possible reason was most of the patients in advanced Wagner stage were developed gangrene.

Peripheral neuropathy was another variable that predicts of amputation in diabetic foot ulcer patients. Diabetic patients who had neuropathy were 1.6 times more likely to be amputated as compared to diabetic patients without neuropathy. This result was consistent with the studies conducted in Germany and Gondar [19,23]. This might due to peripheral neuropathy exposes the patient for the infection of their feet as a result of the increase in the duration of pressure over the diabetic foot. Additionally, higher blood glucose level can result in damage of peripheral nerves which increase the risk of amputation.

The most commonly prescribed individual antibiotics in NRH during the study period was cloxacillin 56(34.15%) followed by metronidazole 43(26.22%) and ceftriaxone 33(20.12%). Study in UK by Wong ML and Coppini DV showed that the most commonly prescribed antibiotics were cefradine, clindamycin, and ciprofloxacin [25]. However, the study done in Sweden showed that metronidazole (56%) and ciprofloxacin (54%) were the most commonly used, followed by flucloxacillin (40%) and cefadroxil (31%) [26]. Additionally, the study done in Switzerland by pittet D also showed that the antibiotics most commonly used included semi-synthetic penicillins, second and third-generation cephalosporins and fluoroquinolones [27]. The variety of individual antibiotic use in a variety of settings was mostly due to the etiologic agent identified, patient condition, availability of the drugs, and preference of the physicians.

The outcome of DFU was strongly associated with inappropriate antibiotics given to treat diabetic foot infection. Diabetes foot ulcers who had taken inappropriate antibiotics were 2.5 more times to be amputated than diabetic foot ulcer which had been treated with appropriate antibiotics. This was correlated with the study conducted in UK in which the amputation rate dropped from about 70% to about 30% with appropriate antibiotic therapy [28]. In our study area, about half of the antibiotics were prescribed inappropriately. Therefore, because of excessive and inappropriate use of antibiotics for treating diabetic foot infections, treatment failure and the risk of amputation increased. Inappropriate antibiotics prescription results in the risk of development of resistant pathogens.

The duration of diabetes prior to presentation didn't affect the outcome of diabetic foot ulcers. Previous studies done in Germany, Pakistan, Jamaica, Khartoum and Arbaminch have demonstrated the inhibitory effects of diabetes on wound healing but the duration of diabetes independently may not be as important as overall blood glucose control (which was not looked at in this study) [13,21,[29], [30], [31]].

Diabetic patients who lived in rural areas often walk with bare feet. This may expose their feet to be injured and may resulted in infections. Despite this, most of the patients in our study area were come from urban and the place of the residence had no significant associations with the outcomes of DFU. However, previous studies conducted in Pakistan, Arbaminch and Gondar had demonstrated that DFU significantly associated with the rural residence of the patients [23,29-30].

From the total diabetic foot ulcer patients, 35(30.43%) were amputated and from those amputated 20(57.14%) and 15(42.86%) were undergone minor and major amputations, respectively. This figure was comparable with the study done in Tanzania and Pakistan [12,30]. However, lower than the study done in Singapore [32]. Additionally, the finding was higher than the study done in university of Malta [33] and Alfayha teaching hospital, Iraq [34]. This was due to the differences in quality of diabetic foot care and the difficulty of obtaining consent for major or even minor surgery that required amputation of an affected limb. The reason for this reluctance lies in part in cultural factors where the loss of limb may be considered worse than loss of life.

### 4.1 Strength and limitation of the study

As a strength, the study was a prospective observational design that helps as baseline information for other researchers. As limitations, fasting plasma glucose was used to assess the adequacy of glycemic control instead of glycosylated hemoglobin (HbA1c) due to availability problems. Additionally, culture and sensitivity tests were not done to identify specific strains of the pathogens. Further, in our country, there was no guideline on antibiotics management of diabetic foot ulcer. As a result, the appropriateness of antibiotics was identified based on standard guidelines of Infectious Diseases Society of America (IDSA) for diagnosis and treatment of diabetic foot infection. Finally, the follow-up period was short, thus failing to take into account any non-healing ulcers resulting in amputation after three months. After patients were discharged we followed up patients by telephone, not by face to face interview thus the accuracy of these self-reported events needs to be evaluated.

## 5 Conclusion

Blood glucose level, Higher BMI (overweight and obesity), inappropriate antibiotics given, neuropathy, and advanced grade of diabetic foot ulcer were factors that predicted the amputation among DFU patients. The rate of amputation of the DFU was found to be high in which most of the patients were amputated below the ankle. The most commonly prescribed antibiotics for treating diabetic foot ulcer was cloxacillin and about half of antibiotics were prescribed inappropriately.

To reduce the associated unwelcomed effects of DFU, especial emphasis should be given for patients having neuropathy and advanced grade of diabetic foot ulcer. To minimize the risk of developing DFU, health educators should emphasize the benefit of weight reduction and managing hyperglycaemia. Additionally, laboratory services should be strengthened like culture and sensitivity tests to identify the specific strain of the pathogen for definitive treatment. Through these prescribers should have to minimize empiric antibiotics prescribing to the possible level. Despite the mortality that was not reported in our study, previous studies found as a primary outcome for DFU. Therefore, we recommend further research to identify mortality rates and associated factors.

## Ethical approval

Ethical clearance was obtained from the Institutional Review Board (IRB) of Jimma University, Institute of health with reference number of IHRPGC/104/208.

## Sources of funding

This work was funded by Jimma University. The funding body did not have any role in study design, data collection, data analysis, interpretation of data or in writing the manuscript.

## Author contribution

FB contributes in the proposal preparation, study design, analysis and writes up of the manuscript. LC contributed to the design of the research protocol. GF contributed to analysis and edition of the manuscripts. KB made a substantial contribution to the local implementation of the study. All authors read and approved the final version of the manuscript and agree to be accountable for all aspects of the work in ensurinVg that questions related to the accuracy or integrity of any part of the work are appropriately investigated and resolved.

## Research registry number

Name of the registry: RESEARCH REGISTRY, https://www.researchregistry.com.

Unique Identifying number or registration ID: researchregistry5237.

Hyperlink to the registration (must be publicly accessible):

https://www.researchregistry.com/register-now#home/registrationdetails/5d70f2520791fb0011b79e9f/

## Guarantor

Firomsa Bekele.

## Provenance and peer review

Not commissioned, externally peer reviewed.

## Consent for publication

Not applicable. No individual person's personal details, images or videos were used in this study.

## Availability of data and materials

The datasets used during the current study is available from the corresponding author on reasonable request.

## Declaration of competing interest

The authors declared that they had no competing interest.

## Abbreviations

AOR: Adjusted odds ratio
BMI: Body mass index
CI: Confidence interval
COR: Crude odds ratio
DFU: Diabetic foot ulcer
DM: Diabetes mellitus
IDSA: Infectious diseases society of America
NGO: Non-governmental organization
NRH: Nekemte referral hospital
PAD: Peripheral arterial disease
QOL: Quality of life
SD: Standard deviation
SPSS: Statistical package for social sciences
SSTI: Skin and soft tissue infections
